# Crucial Roles of miR-625 in Human Cancer

**DOI:** 10.3389/fmed.2022.845094

**Published:** 2022-03-04

**Authors:** Menggang Zhang, Fei Xiong, Shuijun Zhang, Wenzhi Guo, Yuting He

**Affiliations:** ^1^Department of Hepatobiliary and Pancreatic Surgery, The First Affiliated Hospital of Zhengzhou University, Zhengzhou, China; ^2^Key Laboratory of Hepatobiliary and Pancreatic Surgery and Digestive Organ Transplantation of Henan Province, The First Affiliated Hospital of Zhengzhou University, Zhengzhou, China; ^3^Open and Key Laboratory of Hepatobiliary and Pancreatic Surgery and Digestive Organ Transplantation at Henan Universities, Zhengzhou, China; ^4^Henan Key Laboratory of Digestive Organ Transplantation, Zhengzhou, China

**Keywords:** miR-625, cancer, proliferation, therapeutic target, ceRNA

## Abstract

Genetic and epigenetic characteristics are core factors of cancer. MicroRNAs (miRNAs) are small non-coding RNAs which regulate gene expression at the post-transcriptional level *via* binding to corresponding mRNAs. Recently, increasing evidence has proven that miRNAs regulate the occurrence and development of human cancer. Here, we mainly review the abnormal expression of miR-625 in a variety of cancers. In summarizing the role and potential molecular mechanisms of miR-625 in various tumors in detail, we reveal that miR-625 is involved in a variety of biological processes, such as cell proliferation, invasion, migration, apoptosis, cell cycle regulation, and drug resistance. In addition, we discuss the lncRNA-miRNA-mRNA and circRNA-miRNA-mRNA networks and briefly explain the specific mechanisms of competing endogenous RNAs. In conclusion, we reveal the potential value of miR-625 in cancer diagnosis, treatment, and prognosis and hope to provide new ideas for the clinical application of miR-625.

## Introduction

Cancer is a severe health problem worldwide given its increasing incidence and high mortality (1). According to reports, in 2018, there were 9.6 million cancer-related deaths and 18.1 million new cancer cases all over the world (2). Isabelle Soerjomataram and Freddie Bray recently pointed out that the number of cancer patients worldwide is predicted to increase in the next 50 years. It is predicted that 34 million new cancer cases will be diagnosed by 2070, double the number in 2018 (3). Although the understanding of cancer biology is increasing, and efforts are being made to develop more effective and targeted diagnosis, treatment, and prevention strategies, cancer is still the main cause of severe social pressure and a substantial economic burden (4). Current research focuses on finding new diagnostic and prognostic biomarkers and potential molecular targets.

In recent years, evidence has shown that non-coding RNAs, as epigenetic factors, are important in the occurrence and development of cancer, including microRNA (miRNA), long non-coding RNA (lncRNA), and circular RNA (circRNA). Among them, miRNAs are the most studied (5–7). miRNAs, with about 22 nucleotides in length, control numerous biological processes, including proliferation, differentiation, and apoptosis (8–11). As a kind of non-coding RNA, miRNAs mainly interact with complementary sequences in the 3′-untranslated regions (3′-UTRs) of corresponding mRNAs through their seed regions and play a role in inhibiting gene expression at the post-transcriptional level (12, 13). Since a single miRNA can interact with hundreds of mRNAs simultaneously, aberrant miRNA expression is involved in the occurrence and development of numerous diseases, especially cancer (14). The expression of most miRNAs is decreased in cancers; for example, miRNA-4317 is low in gastric cancer (GC) and exerts a tumor suppressor effect through inhibiting cell proliferation by targeting ZNF322 (15). The expression of miR-186 is reduced in hepatocellular carcinoma (HCC), and it can inhibit the occurrence and development of HCC by targeting yes-associated protein 1 (YAP1) (16). miR-186 is also expressed at low levels in breast cancer (BC), and low expression of miR-186 is closely associated with the poor prognosis of BC (17). Some miRNAs are increased in cancers; for example, miR-106b-5p is highly expressed in glioma, non-small-cell lung cancer (NSCLC), HCC, and other tumors (18–21). miR-140-5p is upregulated in renal cell carcinoma and promotes tumor cell progression through the miR-140-5p/KLF9/KCNQ1 axis (22).

miR-625 is a recently discovered miRNA that is widely involved in countless human diseases, including cancer. miRNAs are a group of non-coding RNA with a length of ~22 nucleotides (23–25). They guide the RNA-induced silencing complex (RISC) to degrade mRNA by base pairing with target gene mRNAs, thus inhibiting the expression of the target genes (26, 27). Single-stranded RNA monomers (pre-miRNAs) of ~70–100 bases in size and have a hairpin structure (28). These pre-miRNAs are processed by Dicer enzyme digestion to form mature miRNAs (28–30). For some miRNA precursors, the two separate arms target different sites and comprise the functional mature miRNA; these arms are represented by the notation−5p and−3p, as processed from the 5′ end arm and the 3′ end arm, respectively (e.g., miR-625-5p or miR-625-3p) (28, 31).

In this review, we comprehensively summarize the expression and functions of miR-625 in a variety of cancer types and its underlying mechanisms. Importantly, we will discuss the lncRNA-miRNA-mRNA and circRNA-miRNA-mRNA networks and briefly discuss the specific mechanism of competing endogenous RNAs (ceRNAs). Overall, this article reveals the powerful potential of miR-625 as a cancer biomarker and therapeutic target and aims to provide some suggestions for further basic research or clinical applications.

## miR-625 in Cancers

### Aberrant Expression of miR-625 in Cancers

Recently, many studies have shown that miR-625 expression is aberrant in tumor tissues compared to nontumor tissues. Researchers often use quantitative real-time RT-PCR (qRT-PCR) to detect the expression of miRNAs in the tissues and plasma of cancer patients (32, 33). Furthermore, they also perform further verification at the cellular level. We found that miR-625 expression was decreased in most cancers and increased in a small number of cancers. In malignant pleural mesothelioma (MPM) (34, 35) and thyroid cancer (TC) (36, 37), miR-625 was found to be upregulated. It is downregulated in bladder cancer (38), nasopharyngeal carcinoma (NPC) (39), lung cancer (40–46), HCC (47), cervical cancer (CC) (48, 49), osteosarcoma (50), melanoma (51–54), laryngeal squamous cell carcinoma (LSCC) (55), acute myeloid leukemia (AML) (56), BC (57–59), glioma (60, 61), esophageal cancer (EC) (62–64), clear cell renal cell carcinoma (65), GC (66–70), and pancreatic ductal adenocarcinoma (PDAC) (71). In colorectal cancer (CRC), the expression of miR-625 seems to vary, being either high or low. Lou et al. found that miR-625 expression was low in cancer tissues compared with adjacent normal tissues (72). Shang et al. also revealed that miR-625-5p was downregulated in CRC (73). In addition, other researchers found that miR-625-3p expression was increased in CRC (74–76). Furthermore, Rasmussen reported that miR-625-3p was not dysregulated between cancer and non-cancer samples (77). These conflicting findings may be attributed to the different action sites of miR-625 and differences in sample size and source. The expression status of and other parameters related to miR-625 in different cancers are depicted in Table 1.

**Table 1:** The expression, clinical significance, function, and mechanism of miR-625 in different cancers.

**Cancer types**	**miRNA**	**Expression**	**Clinicopathologic features**	**Prognosis**	**Target gene**	**Pathway**	**Functions**	**References**
Bladder cancer	miR-625-5p	Down	/	Poor	Runx1t1, TCF4, RBM24	/	Inhibits proliferation	(65)
Nasopharyngeal carcinoma	miR-625	Down	/	/	NUAK1	/	Inhibits proliferation, migration, invasion, metastasis, induces apoptosis	(37)
Non-small cell lung cancer	miR-625-3p	Down	/	/	AXL	TGF-β/Smad pathway	Attenuates gefitinib resistance	(39)
	miR-625-5p	Down	/	/	PCNA, cyclin D1, cyclin E, p16, p21	/	Inhibits proliferation, induces apoptosis	(40)
	miR-625	Down	/	/	Resistin	Resistin/PI3K/AKT/Snail pathway	Inhibits proliferation, invasion, migration, EMT	(41)
	miR-625	Down	Tumor size, lymph node metastasis, TNM stage	Poor	HOXB5	Wnt/β-catenin pathway	Inhibits proliferation, migration, invasion, metastasis, induces apoptosis	(42)
Lung adenocarcinoma	miR-625-5p	Down	/	/	PKM2	/	inhibits proliferation, invasion, migration	(43)
	miR-625-5p	Down	/	/	CPSF7	/	Inhibits proliferation, migration, invasion, induces cell cycle arrest and apoptosis	(44)
Hepatocellular carcinoma	miR-625	Down	Higher lymph node and distance metastasis, the presence of portal venous invasion, TNM stage	Poor	IGF2BP1	IGF2BP1/PTEN pathway	Inhibits migration, invasion, metastasis	(45)
Cervical cancer	miR-625-5p	Down	/	Poor	NFKB1, cyclin D1, CDK4	NF-κB Signaling	Inhibits proliferation	(46)
	miR-625-5p	Down	/	/	LRRC8E	/	Inhibits proliferation, migration, invasion, metastasis, induces cell cycle arrest and apoptosis	(47)
Osteosarcoma	miR-625	Down	/	/	YAP-1	/	Inhibits proliferation and invasion	(48)
Melanoma	miR-625-5p	Down	Tumor stage, lymph node metastasis	Poor	IGF-1R	/	Inhibits proliferation, migration, invasion, induces cell cycle arrest and apoptosis, attenuates cisplatin resistance	(49)
	miR-625	Down	/	/	YY1	/	/	(50)
	miR-625-5p	Down	TNM stage, tumor size, and poor differentiation	/	PKM2	/	Inhibits proliferation and glycolysis	(51)
	miR-625	Down	/	/	SOX2	/	Inhibits proliferation, clonogenicity, migration, invasion	(52)
Laryngeal squamous cell carcinoma	miR-625	Down	Advanced clinical stage, lymph node metastasis	/	SOX4	/	Inhibits proliferation, migration, invasion, EMT	(53)
Acute myeloid leukemia	miR-625-5p	Down	/	/	SOX12	/	Inhibits proliferation, induces apoptosis	(54)
	miR-625	Down	/	/	/	Wnt/b-catenin signaling	Inhibits proliferation, migration	(78)
Thyroid cancer	miR-625-3p	Up	/	/	MMP-9	PI3K/AKT and MEK/ERK signaling pathways	Promotes migration and invasion, induces apoptosis	(34)
	miR-625-3p	Up	/	/	AEG-1	Wnt/β-catenin and JNK pathways	Promotes proliferation, migration, invasion	(35)
Colorectal cancer	miR-625-3p	Up	/	/	MAP2K6	MAP2K6-p38 signaling	Induces oxaliplatin resistance	(72)
	miR-625-5p	Down	/	/	LASP1	/	/	(71)
	miR-625-3p	Up	/	/	/	/	Induces oxaliplatin resistance	(73)
	miR-625	Down	Advanced lymph node metastasis, liver metastasis, poor overall survival	Poor	/	/	Inhibits migration, invasion, metastasis	(70)
	miR-625-3p	/	/	/	/	/	Induces oxaliplatin resistance	(75)
	miR-625-5p	Down	/	/	ZEB2	/	/	(55)
Breast cancer	miR-625	Down	/	/	HMGA1	/	/	(56)
	miR-625	Down	Estrogen receptor, human epidermal growth factor receptor 2, clinical stage	Poor	HMGA1	/	Inhibits proliferation and migration	(57)
	miR-625-5p	Down	/	/	/	/	Inhibits migration and invasion	(58)
Glioma	miR-625	Down	/	/	AKT2	/	Inhibits proliferation, colony formation, induces G0/ G1 arrest, increases the chemosensitivity	(59)
	miR-625	Down	/	/	/	/	Inhibits proliferation, migration, invasion,	(60)
Esophageal cancer	miR-625	Down	Tumor depth, tumor stage, metastasis	/	SOX2	/	Inhibits proliferation, invasion, metastasis.	(61)
	miR-625	Down	Lymph node metastasis, distant metastasis, tumor differentiation, advanced TNM stage	Poor	/	/	/	(62)
Esophageal squamous cell carcinoma	miR-625	Down	/	/	CCND1	/	Inhibits proliferation, colony formation, migration, invasion, induces G0/G1 arrest and apoptosis	(63)
Clear cell renal cell carcinoma	miR-625-5p	Down	/	/	STAT3	/	Inhibits proliferation, migration, invasion,	(64)
Gastric cancer	miR-625-3p	Down	Lymph node or distant metastasis	Poor	EZH2	/	Inhibits proliferation and metastasis	(65)
	miR-625-5p	Down	/	/	NFIX	/	Inhibits proliferation, reduces apoptosis	(66)
	miR-625	Down	/	/	ALDH1A1	/	Induces apoptosis, reverses multidrug resistance	(67)
	miR-625	Down	Lymph node metastasis	/	ILK	/	Inhibits invasion and metastasis	(68)

### Non-Small-Cell Lung Cancer (NSCLC)

Lung cancer is one of the most malignant tumors in the world, and it is also a leading cause of cancer-associated deaths among males and females (79, 80). Lung cancers are categorized into two major histological types: NSCLC and small-cell lung cancer (81). NSCLC accounts for approximately 85% of all lung cancers (82, 83). An increasing number of studies have revealed the anti-cancer effect of miR-625 in NSCLC. Xiaoxia Tan found that low expression of miR-625 was related to advanced clinical characteristics and poor overall survival (OS) of patients with NSCLC (44). In addition, *in vitro* and *in vivo* studies have proven that miR-625 suppresses cell proliferation, migration and invasion and induces apoptosis in NSCLC. Further experiments proved that miR-625 inactivated the Wnt/β-catenin pathway by targeting Homeobox B5 (HOXB5), thereby exerting a tumor suppressor effect in NSCLC. Homeobox (HOX) genes are a family of transcription factors (84), and HOXB5 belongs to the HOX gene family (85, 86). Zhang et al. previously reported that knockdown of HOXB5 inhibited β-catenin expression and its downstream targets cyclin D1 and c-Myc in A549 cells (87). HOXB5 significantly promoted NSCLC cell growth, invasion, metastasis, and epithelial-mesenchymal transition (EMT), partly through the Wnt/β-catenin signaling pathway. Another study revealed that miR-625 suppresses NSCLC cell metastasis by obstructing the resistin/PI3K/AKT/Snail pathway and by decreasing EMT (43). Lung adenocarcinoma (LAC) is the most common histological subtype of NSCLC (88). Xue and Yang et al. reported that miR-625 was involved in the process by which lncRNAs promoted the progression of LAC (45, 46). Interestingly, miR-625 shows obvious anti-inflammatory effect in both lung injury model and asthma model, through completely different molecular mechanisms (89, 90). And there is an inseparable relationship between inflammation and tumorigenesis. This also explains anti-tumor effect of miR-625 in the deeper mechanism.

**Figure 1:**
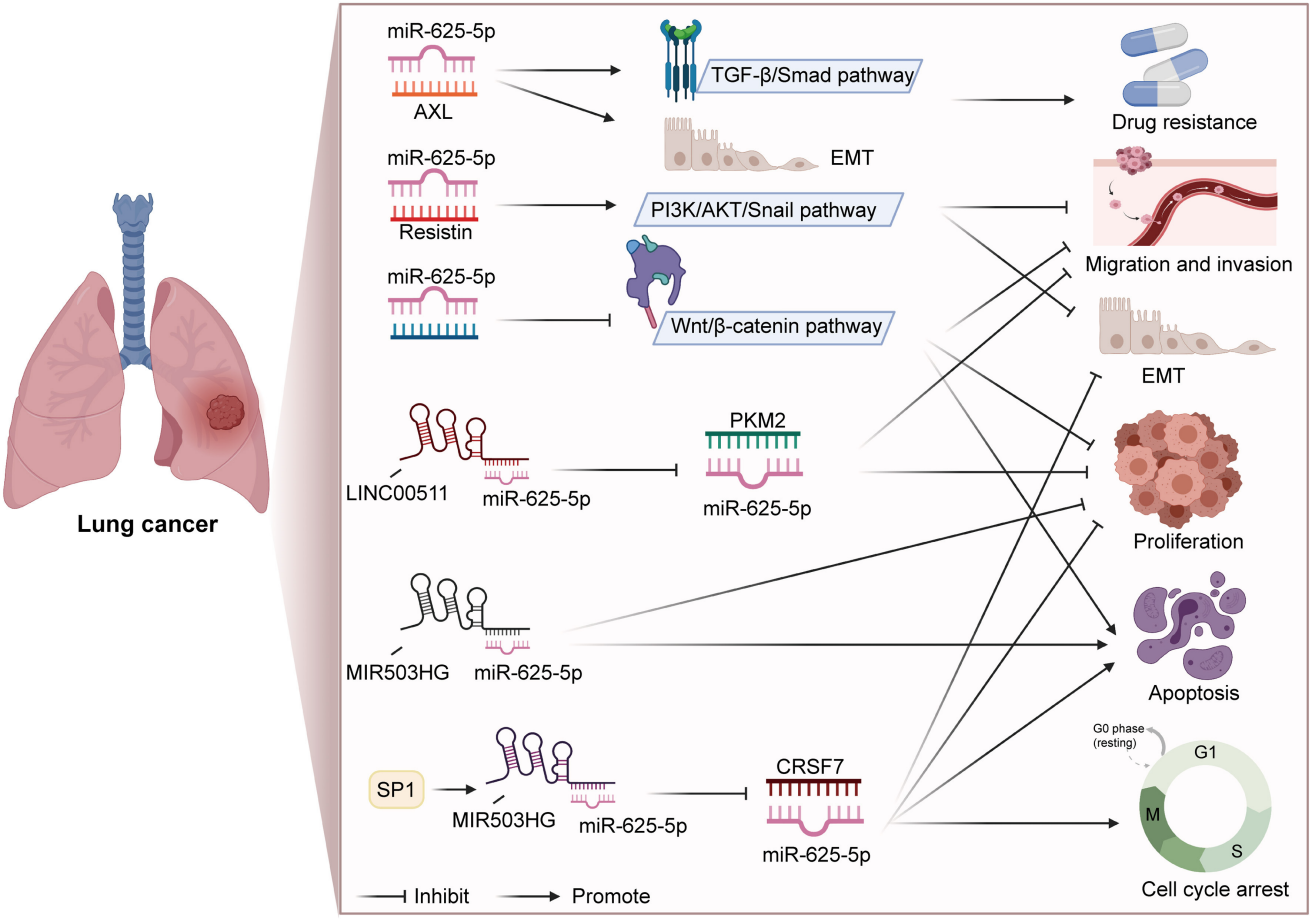
Summary diagram of the roles and mechanisms of miR-625 in lung cancer (Created with BioRender.com).

In addition to participating in the regulation of a series of biological processes, miR-625 is also closely related to drug resistance and disease diagnosis. Du et al. found that miR-625-3p overexpression reversed gefitinib resistance (41). At present, gefitinib is the preferred treatment NSCLC patients with for epidermal growth factor receptor (EGFR) mutation (91, 92). Mechanistically, miR-625-3p overexpression was found to inhibit the EMT induced by TGF-β1 and enhance gefitinib sensitivity by targeting AXL (41). Roth et al. conducted a blood-based microRNA expression profile analysis, and the results showed that miR-625^*^ was lower in NSCLC patients than in healthy controls or those with benign disease (40). In addition, the levels of miR-625^*^ were noticeably lower in patients who smoked and large-cell lung cancer patients than in nonsmoking patients and adenocarcinoma patients, respectively. Therefore, miR-625^*^ expression can be used as a blood-based marker for disease diagnosis. Figure 1 summarizes the regulatory mechanism of miR-625 in lung cancer. These results reveal that miR-625 is a novel diagnostic and prognostic biomarker and treatment target for lung cancer.

### Cervical Cancer (CC)

Cervical cancer is the fourth most common malignant tumor in women and is the main cause of gynecological tumor-related death in the world (93, 94). The most common predisposing cause of CC is high-risk human papillomavirus (HPV) infection (95, 96). Persistent HPV infection can lead to chronic inflammation, thereby causing cervical intraepithelial neoplasia (CIN) as well as cervical cancer (97). Although great progress has been made in the prevention, diagnosis, and treatment of CC, the OS rate of patients is still unsatisfactory, partly due to its late detection and late recurrence (98, 99). In recent years, there have been many studies on miRNAs in CC. Researchers have tried to find new tumor markers for the early diagnosis of CC to improve the disease detection rate and reduce the mortality rate. Li et al. found that miR-625-5p expression was significantly low in CC tissues and cell lines (48). The downregulation of miR-625-5p is linked to unfavorable clinical prognosis of CC patients. Overexpression of miR-625-5p suppresses cell proliferation in cervical carcinoma. Another study confirmed that the LINC00958/miR-625-5p/LRRC8E axis participated in CC cell proliferation and metastasis (49).

### Melanoma

Melanoma arises from the deterioration of melanocytes located in the basement of the epidermis (100). It is the most aggressive form of skin cancer (101, 102). Pyruvate kinase (PK) participates in the transformation of phosphoenolpyruvate to pyruvate and is a rate-limiting enzyme in the last step of the glycolysis process (103, 104). Pyruvate kinase m2 (PKM2) is an alternative splice variant of PK (105). Evidence from clinical *in vitro* and *in vivo* studies shows that PKM2 is an important molecule in processes related to progression of cancer, such as glucose metabolism and apoptosis (78, 106, 107). Zhang et al. found a negative correlation between the expression of miR-625-5p and PKM2 in clinical melanoma specimens (53). They further revealed that miR-625-5p inhibited the proliferation, lactic acid production, ATP production, and glucose consumption of melanoma cells by targeting PKM2. Another study also revealed a tumor suppressor role of miR-625 in melanoma. miR-625 can inhibit melanoma cell proliferation, wound healing, migration, and tumorigenicity. Mechanistically, miR-625 plays a role at least in part by inhibiting SOX2 (54). In addition, a relationship between the expression of miR-625 and clinical characteristics has been noted. The results show that miR-625 is associated with tumor size, lymph node metastasis, TNM stage, and differentiation (51, 53).

### Acute Myeloid Leukemia (AML)

AML is a malignant hematopoietic system disease with high morbidity and mortality (108, 109). Gain-of-function assays suggested that after transfection with miR-625-5p, the proliferation of U937 and HL60 cells was significantly reduced, whereas the miR-625-5p + SOX12 group had the opposite pattern. Therefore, miR-625-5p can regulate AML cell growth by targeting SOX12 (56). Ma et al. also pointed out that miR-625 participated in the occurrence and development of AML through Wnt/β-catenin signaling (110).

### Thyroid Cancer (TC)

Although miR-625 is down-regulated in most human diseases, its expression in TC is increased (36, 37). Fang et al. investigated the function, molecular mechanism, and signaling pathways of miR-625 in TC (37). The researchers found that miR-625-3p overexpression promotes cancer cell proliferation, migration, and invasion by upregulating the expression of astrocyte elevated gene 1 (AEG-1). Moreover, overexpression of AEG-1 promotes the activation of Wnt/β-catenin and JNK pathways (37). Another study showed that icariin (ICA) exerted tumor inhibitory effect by blocking TC cell proliferation, and metastasis by suppressing miR-625-3p. In addition, ICA can also inactivate the PI3K/AKT and MEK/ERK signaling pathways by regulating miR-625-3p in CC cells (36).

### Colorectal Cancer (CRC)

An increasing number of researchers are exploring the link between miRNAs and CRC and possible mechanistic targets. miR-625-3p was found to play an oncogenic role in CRC: it could promote cell migration and invasion and induce oxaliplatin resistance (74–76). The SCAI/E-cadherin/MMP-9 pathways (76) and MAP2K6-p38 signaling are involved in this process (74). Lou et al. revealed the tumor suppressor effect of miR-625 in CRC (72). The researchers found that miR-625 was obviously downregulated in CRC tissues and cell lines. The expression of miR-625 had an inverse relationship with the lymph node metastasis and liver metastasis status. The univariate and multivariate analysis results both showed that miR-625 could be used as an independent prognostic factor for CRC. *In vivo* and *in vitro* experiments also revealed that miR-625 inhibited invasion and migration. Similarly, another study reported that miR-625-5p was involved in the inhibition of CRC development (73).

### Breast Cancer (BC)

BC is the most frequently diagnosed malignancy in females worldwide (80, 111). At present, the main treatment strategies include surgical resection, radiotherapy and chemotherapy, hormone therapy, and targeted biological therapy (112). Although the prognosis of BC patients has improved, BC is still the main cause of cancer-related deaths in women (113, 114). Zhou and his colleagues conducted clinical studies and *in vitro* cell function experiments, revealing the clinical value of miR-625 in BC. They found that miR-625 expression was decreased in BC and related to poor outcomes. The decreased expression level of miR-625 was closely associated with estrogen receptor (ER) and human epidermal growth factor receptor 2 (HER2) expression and clinical stage (59). In addition, miR-625 inhibits cell proliferation and migration by regulating HMGA1, its downstream target. Mechanistically, HMGA1 transfers YAP to the nucleus by regulating the activity of cyclin E2, thereby promoting cell migration and invasiveness (115). Wu and Qi et al. also reported that miR-625 exerted a tumor suppressor effect in BC (57, 58).

### Glioma

Glioma accounts for ~80% of brain malignancies and is the most common intracranial tumor (116–118). It is characterized by high malignancy, strong invasiveness, and poor prognosis (119, 120). Studies have found that circDENND2A promotes hypoxia-induced migration and invasion of U87MG and A172 cells. However, miR-625-5p inhibited these effects and had a tumor suppressor effect in gliomas (60). In addition, Zhang and his colleagues conducted a series of studies to explore the role of miR-625 in glioma. They used BALB/c nude mice to conduct *in vivo* experiments, and the results showed that miR-625 inhibited tumor growth and angiogenesis. *In vitro* functional experiments revealed that miR-625 suppressed glioma cell proliferation and colony formation and induced G0/G1 arrest, thereby influencing cell cycle progression (61). Moreover, miR-625 enhanced temozolomide (TMZ) chemosensitivity by targeting AKT2. Drug resistance has always been a difficult problem in cancer treatment (121). Therefore, miR-625 can be used as a treatment target for glioma.

### Esophageal Cancer (EC)

miR-625 has been reported to be downregulated in EC. Low miR-625 expression was significantly correlated with tumor stage, tumor depth, and metastasis. Functionally, miR-625 inhibits tumor cell proliferation, migration, and metastasis but does not affect apoptosis. Mechanistically, miR-625 works by directly binding to the 3′-UTR of SOX2 (63). Chuan Li evaluated the correlation of miR-625 expression with clinicopathological features in 169 pairs of ESCC tissues and adjacent non-tumor tissues (64). Low miR-625 expression is closely linked to lymph node and distant metastasis, poor tumor differentiation, and advanced TNM stage. Moreover, the 5-year OS rate in the low expression group is 38.1%, compared with 68.8% in the high expression group. All these results suggest that down-regulation of miR-625 may serve as a novel biomarker to predict tumor progression and poor prognosis in EC patients.

### Gastric Cancer (GC)

Accumulating evidence has shown that miR-625 is involved in many processes in the development and progression of GC. Wang et al. reported the tumor suppressor effect of miR-625 in GC (70). In clinical application studies, it was found that low expression of miR-625 was related to lymph node metastasis. In mechanistic studies, the results showed that miR-625 was an important regulator of the migration and invasion potential of GC cells. miR-625 inhibits the migration and invasion of cells by regulating ILK. Furthermore, miR-625 is also involved in influencing multidrug resistance (MDR). According to a study report, miR-625 reverses MDR in GC cells by inhibiting ALDH1A1 (69). Consistent with previous studies, Li and colleagues also found that low miR-625-3p had a close relationship with lymph node and distant metastasis. A Kaplan-Meier survival curve analysis indicated that low miR-625-3p expression was remarkably associated with poor prognosis in GC patients. In addition, miR-625-3p was found to regulate the proliferation and migration of GC cells through the inhibition of EZH2 expression (67).

### Other Cancers

In bladder cancer (38), NPC (39), HCC (47), osteosarcoma (50), LSCC (55), ccRCC (65), and PDAC (71), miR-625 plays a protective role. Up-regulation of miR-625 can affect tumor progression through different mechanisms of action. Zhou et al. found that a decrease in miR-625 was obviously associated with lymph node and distant metastasis, the presence of portal venous invasion, advanced TNM stage, and unfavorable OS (47). Further investigation revealed that the miR-625/IGF2BP1/PTEN axis participated in the occurrence and development of HCC. Moreover, we know that liver cirrhosis is an important risk factor for HCC. The latest research shows that the expression level of miR-625 is related to the levels of alanine aminotransferase (ALT) and aspartate aminotransferase (AST), and the results of receiver operating characteristic (ROC) analysis show that miR-625 has ideal sensitivity and specificity in the diagnosis of liver cirrhosis (122). Therefore, we have reason to believe that miR-625 will contribute to the early diagnosis of HCC. EMT is a complicated trans-differentiation process that is a hallmark of cancer (123, 124). Cancer cells gain the ability to migrate and invade through this process (125, 126). Li et al. point out that up-regulated miR-625 promoted E-cadherin and inhibited N-cadherin and vimentin, suppressing EMT of LSCC cells (55). Interestingly, through a systematic review and a qualitative meta-analysis, a series of miRNAs have been found which have the potential to diagnose malignant mesothelioma, including miR-625. Although this result was found several years ago and had limitation, it still can confirm the biomarker potential of miR-625 again. In addition, researchers found that miR-625 worked by targeting Sex-determining region Y-box 4 (SOX4). Researchers have found that miR-625-3p could be used as a marker to assess the prognosis of cancer (34, 35).

### ceRNA Networks Related with miR-625

**Figure 2:**
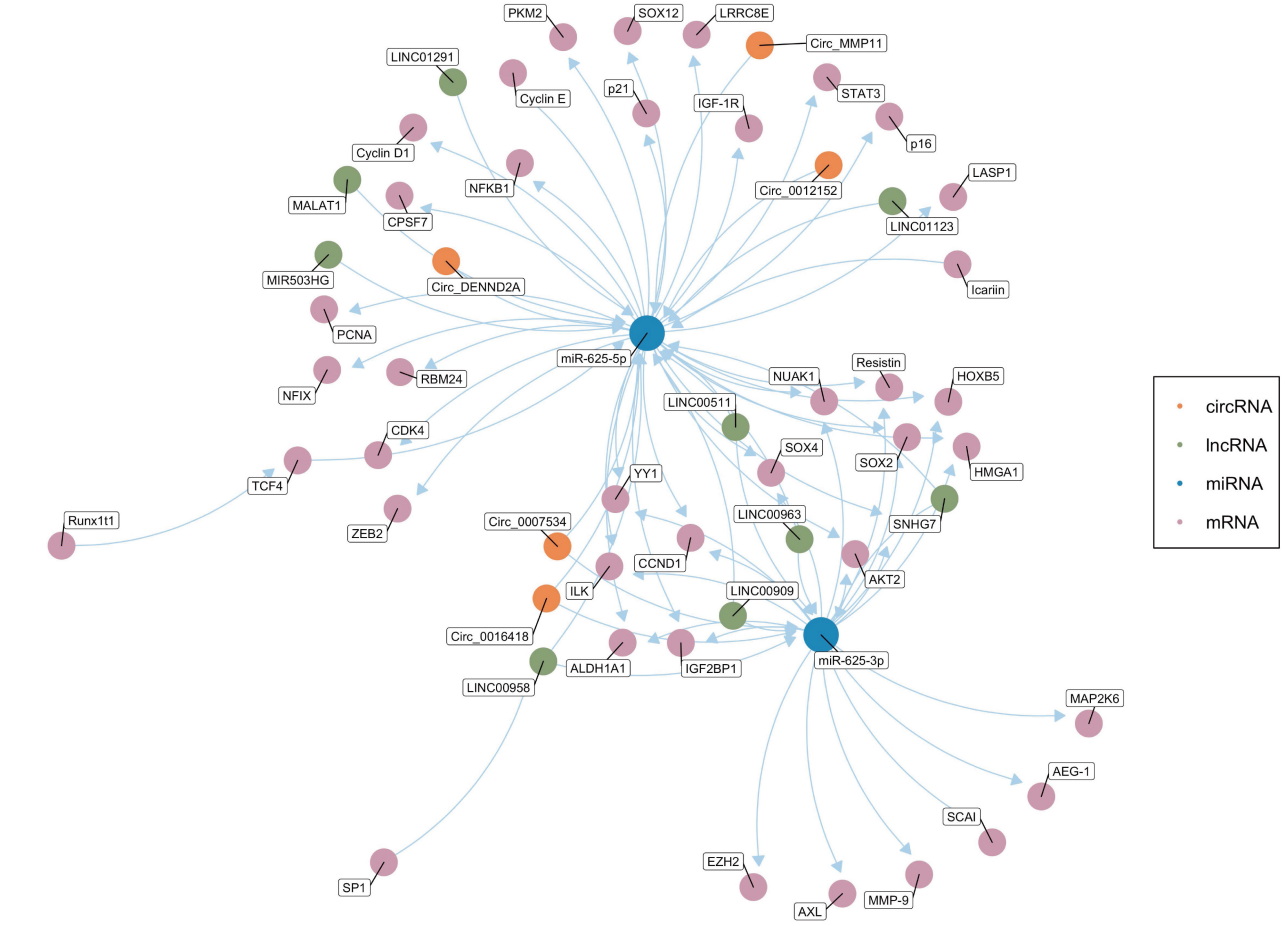
The competing endogenous RNA (ceRNA) network of circRNAs/lncRNAs-miR-625-mRNAs. The green dots represent lncRNAs, and the orange dots represent circRNAs. The blue dots represent miR-625-5p or miR-625-3p. The pink dots represent mRNAs (Created with BioRender.com).

Salmena et al. first proposed the ceRNA hypothesis, which concerns how RNA interacts through microRNA response elements (MREs) (127). As mentioned above, miRNAs mainly inhibit the expression of target genes at the post-transcriptional level (12, 13). ceRNAs can function as natural miRNA sponges and competitively bind to and deactivate miRNAs through MREs, hence influencing the mRNA level of target genes (128–130). Theoretically, any RNA molecule may become an active ceRNA if it shares miRNA binding sites with other RNAs (131). Currently, the most studied ceRNAs are lncRNAs, circRNAs, pseudogenes, and protein-coding transcripts (131–135). Cancer is usually related to abnormal gene expression at the transcriptional and post-transcriptional levels (124). Gene expression is a key determinant of cell phenotype (136). In recent years, studies have found that ceRNAs play an important role in the pathogenesis and development of cancer by affecting the expression of carcinogenic and tumor suppressor genes. Figure 2 shows the ceRNA network of circRNAs/lncRNAs-miR-625-mRNAs clearly and comprehensively.

### circRNAs and miR-625

Table 2 summarizes known circRNA-miR-625-mRNA networks. Y Zou evaluated the expression of circ0016418 in skin melanoma (52). The qRT-PCR results showed that compared with that in adjacent normal tissues, the circ0016418 in melanoma tissues was remarkably higher. Further research revealed that circ0016418 exerted a carcinogenic effect by regulating the miR-625/YY1 axis. Shang et al. found that down-regulation of circ0012152 suppressed proliferation and induced apoptosis of AML cells *via* the miR-625-5p/SOX12 axis (56). In addition, the circMMP11/miR-625-5p/ZEB2 axis is involved in the BC cell proliferation, migration and apoptosis (57). Moreover, circDENND2A promotes the migration and invasion of glioma cells by sponging miR-625-5p (60). Hao et al. found that circ0007534 was significantly related to PDAC stage and lymph node infiltration (71). The results of survival analysis shows that an increase in circ0007534 indicates a poor prognosis. Mechanistically, the carcinogenic function of circ0007534 partly depends on the regulation of miR-625 and miR-892b.

**Table 2:** Summarization of circRNA-miR-625 in human cancers.

**Cancer types**	**CircRNA**	**Expression**	**miRNA**	**References**
Melanoma	CircRNA-0016418	Up	miR-625	(50)
Acute myeloid leukemia	CircRNA-0012152	Up	miR-625-5p	(54)
Breast cancer	CircRNA-MMP11	Up	miR-625-5p	(55)
Glioma	CircRNA-DENND2A	Up	miR-625-5p	(58)
Pancreatic ductal adenocarcinoma	CircRNA-0007534	Up	miR-625	(69)

### lncRNAs and miR-625

Table 3 summarizes known lncRNA-miR-625-mRNA networks. In LAC, ccRCC, and GC, LINC00511 exerts carcinogenic effects by sponging miR-625. Members of the LINC00511/miR-625-5p/PKM2 axis may be helpful therapeutic targets for LAC (45). Similarly, Huanghao Deng pointed out that the LINC00511/miR-625/CCND1 axis participated in ccRCC progression and was a potential therapeutic target (65). LINC00511 promotes GC progression through the miR-625-5p/STAT3 axis and miR-625-5p/NFIX axis (66, 68). In NPC (39), LAC (46), and CC (49), LINC00958 functions as a ceRNA that can promote the growth, migration, and invasion of tumor cells by sponging miR-625. The study found that an increase in circulating miR-625-3p and a decrease in lncRNA GAS5 were significantly related to MPM progression. Reduced GAS5 is significantly associated with shorter OS and progression-free survival. The study also revealed the potential value of GAS5 in patients treated with platinum-adjuvant chemotherapy (34). Dao et al. reported that knockdown of the lncRNA MIR503HG inhibited proliferation and induced apoptosis of NSCLC cells by regulating miR-625-5p and miR-489-3p (42). Ma and his colleagues explored the clinical significance of LINC00909 expression in AML patients. The results showed that LINC00909 was an independent prognostic indicator of OS for AML patients. Further research found that LINC00909 promoted disease progression by regulating the miR-625/b-catenin axis (110). Wang et al. reported that expression of the lncRNA SNHG7 was enhanced in ESCC, promoting ESCC cell proliferation, and metastasis by regulating miR-625 (62). In addition, the lncRNA MALAT1/miR-625-5p/NF-κB, LINC01291/miR-625-5p/IGF-1R, LINC01123/miR-625-5P/LASP1 and LINC00963/miR-625/HMGA1 pathways play important roles in CC, melanoma, CRC, and BC, respectively (48, 51, 58, 73).

**Table 3:** Summarization of lncRNA-miR-625 in human cancers.

**Cancer types**	**LncRNA**	**Expression**	**miRNA**	**References**
Nasopharyngeal carcinoma	LINC00958	Up	miR-625	(37)
Malignant pleural mesothelioma	GAS5	Up	miR-625-3p	(32)
Non-small lung cancer	MIR503HG	Up	miR-625-5p	(40)
Lung adenocarcinoma	LINC00511/LINC00958	Up	miR-625-5p	(43)
Cervical cancer	MALAT1/LINC00958	Up	miR-625-5p	(44)
Melanoma	LINC01291	Up	miR-625-5p	(49)
Acute myeloid leukemia	LINC00909	Up	miR-625	(78)
Breast cancer	LINC00963	Up	miR-625	(56)
Esophageal cancer	SNHG7	Up	miR-625	(60)
Clear cell renal cell carcinoma	LINC00511	Up	miR-625	(63)
Gastric cancer	LINC00511	Up	miR-625-5p	(64)
Colorectal cancer	LINC01123	Up	miR-625-5p	(71)

## Conclusions and Perspectives

**Figure 3:**
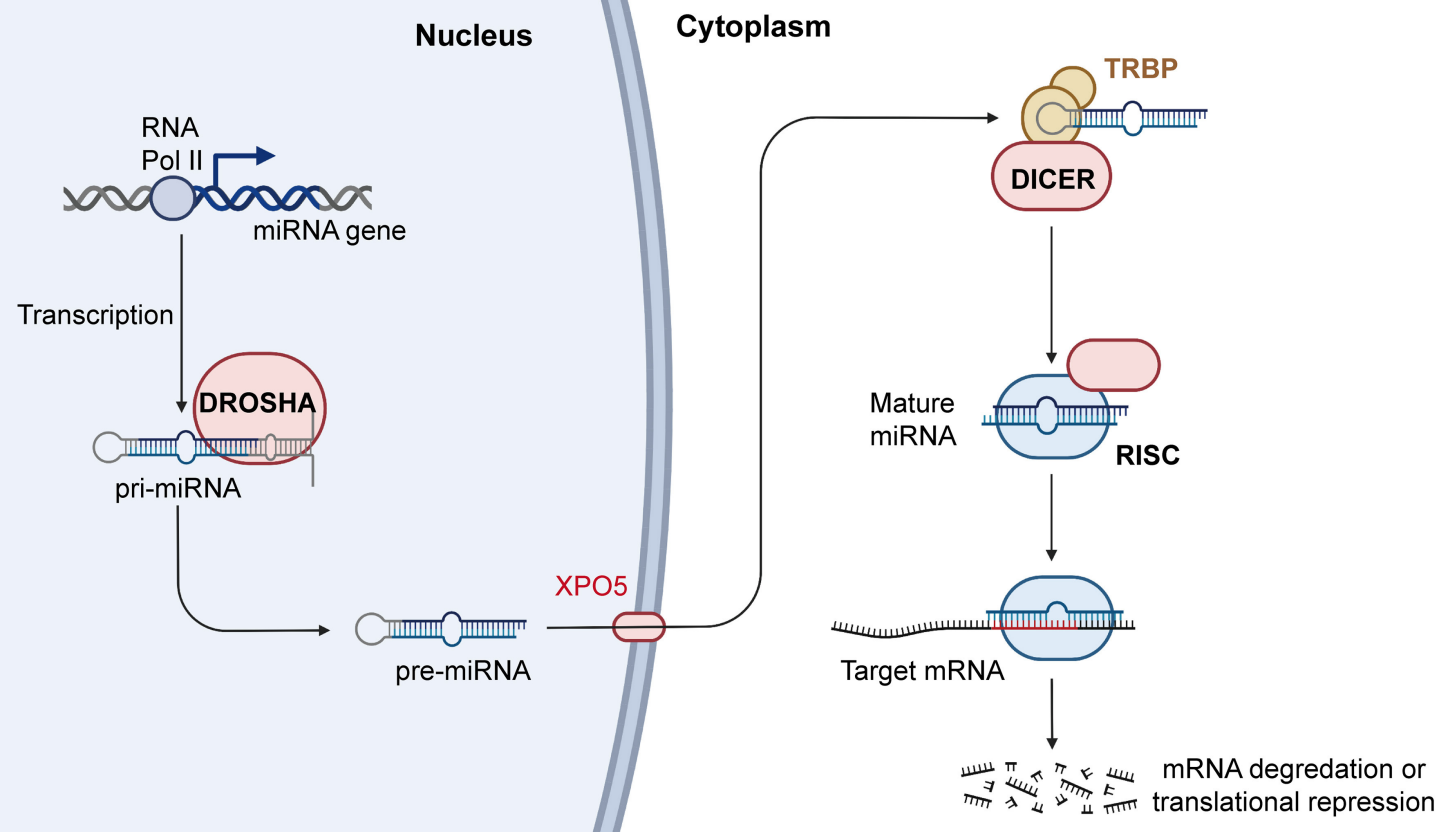
Biogenesis and mechanism of miRNA. RNAPII transcribes miRNA genes into pri-miRNAs, and the pri-miRNAs are cleaved by Drosha into the pre-miRNAs. Pre-miRNAs are transferred from nucleus to cytoplasm with the help of XPO5, and Dicer can process the pre-miRNAs into mature miRNAs. Mature miRNAs then combine with AGO2 to form RISCs, which further play an important role in regulating gene expression. RNAPII, RNA polymerase II; XPO5, exportin-5; AGO2, argonaute RISC catalytic component 2; RISC, RNA induced silencing complex (Created with BioRender.com).

In recent years, increasing evidence has shown that miRNAs participate in the occurrence and development of cancers. miRNAs are abnormally expressed in cancers and are widely involved in a variety of biological processes, including proliferation, migration, invasion, cell cycle regulation, apoptosis, and intracellular metabolism. Some abnormally expressed miRNAs are closely related to clinical features and can also be used as independent markers of disease prognosis. Further understanding the miRNA biogenesis plays a vital role in the follow-up miRNA drug research and exploring the functions of miRNA in the occurrence and development of cancers. Therefore, we summarized the latest miRNA biogenesis process in Figure 3. In addition, miRNAs also play an important role in tumor treatment, especially in drug resistance. For example, Wang et al. found that FER1L4 could suppress miR-106a-5p/miR-372-5p expression, activating the E2F1-mediated NF-κB pathway and thus leading to drug resistance in liver cancer (137). Luo et al. also reported that FOXO3a-miRNA feedback could lead to Herceptin resistance in BC (138). In CRC, it was reported that the HSF1/miR-135b-5p axis could promote oxaliplatin resistance *via* the MUL1/ULK1 pathway (139). Interestingly, chronic accumulation of senescent cells and the concomitant senescence-associated secretory phenotype contribute to tumor microenvironment remodeling. In this process, the levels of a large number of related miRNAs have changed greatly. After measurement the level of miRNAs one by one, it is found that the level of miR-625 continues to decline with cell senescence. The trend of miR-625 in cell senescence is consistent with the trend of miR-625 in tumors (140). So, we can guess that the level of miR-625 may be a part of the tumor microenvironment affecting the occurrence and development of cancer.

There has been increasing research on miR-625. miR-625 can function as a tumor suppressor or a tumor-promoting factor. The abnormal expression of miR-625 in whole blood, plasma, urine, and other samples of tumor patients can be assessed to diagnose cancer with non-invasive methods (34, 35). In fact, according to the existing research progress, we believe that the clinical diagnostic biomarker value of miR-625 will be expected to play in the clinic in the near future. The future research direction should focus on the statistical work before clinical application and clinical application verification. LncRNAs and circRNAs participate in the occurrence and progression of various cancers by regulating miR-625. In addition to ncRNAs, icariin also promotes the development of cancer through the regulation of miR-625 (36). In addition, we believe that miR-625 is an extraordinarily valuable target for cancer treatment, especially in the treatment of drug resistance. Wu et al. found that inhibiting miR-625-5p or up-regulating IGF-1R could offset the regulatory effect of siLINC01291 on the sensitivity of melanoma cells to cisplatin chemotherapy (51). In NSCLC (41), CRC (75), and GC (69), miR-625 has also been reported to affect drug resistance.

In this review, the expression and function of miR-625 in cancer were reviewed, and the related molecular mechanisms were briefly discussed. However, due to the limited number of existing studies and the dual role of miR-625 in the development of some tumors, a large number of basic experiments, animal models, and clinical studies are needed to further reveal and verify its function and clinical significance. Overall, miR-625 is expected to be a promising new target for cancer treatment.

## Author Contributions

WG, YH, and SZ designed and guided the review. MZ wrote the manuscript. YH and FX drew the mechanism diagrams. WG and YH edited and reviewed the manuscript. All authors read and approved the final manuscript.

## Funding

This work was supported by Leading Talents of Zhongyuan Science and Technology Innovation (214200510027), Henan Provincial Medical Science and Technology Research Plan (SBGJ2018002 and SBGJ202102117), Youth Talent Lifting Project of Henan Province (2021HYTP059), Key Scientific Research Project of Henan Higher Education Institutions of China (21A320026), Henan Medical Science and Technology Joint Building Program (LHGJ20210324), Science and Technology Innovation Talents in Henan Universities (19HASTIT003), Outstanding Foreign Scientist Studio in Henan Province (GZS2020004), and the Gandan Xiangzhao Research Fund (GDXZ2022002).

## Conflict of Interest

The authors declare that the research was conducted in the absence of any commercial or financial relationships that could be construed as a potential conflict of interest.

## Publisher's Note

All claims expressed in this article are solely those of the authors and do not necessarily represent those of their affiliated organizations, or those of the publisher, the editors and the reviewers. Any product that may be evaluated in this article, or claim that may be made by its manufacturer, is not guaranteed or endorsed by the publisher.
